# Electrochemical l-Lactic Acid Sensor Based on Immobilized ZnO Nanorods with Lactate Oxidase

**DOI:** 10.3390/s120302456

**Published:** 2012-02-23

**Authors:** Zafar Hussain Ibupoto, Syed Muhammad Usman Ali Shah, Kimleang Khun, Magnus Willander

**Affiliations:** Department of Science and Technology, Campus Norrköping, Linköping University, SE-60174, Norrköping, Sweden; E-Mails: usman.ali@liu.se (S.M.U.A.S.); kimleang.khun@liu.se (K.K.); magnus.willander@liu.se (M.W.)

**Keywords:** zinc oxide nanorods, lactate oxidase enzyme, glutaraldehyde, potentiometric nanostructured biosensor, nanodevices

## Abstract

In this work, fabrication of gold coated glass substrate, growth of ZnO nanorods and potentiometric response of lactic acid are explained. The biosensor was developed by immobilizing the lactate oxidase on the ZnO nanorods in combination with glutaraldehyde as a cross linker for lactate oxidase enzyme. The potentiometric technique was applied for the measuring the output (EMF) response of l-lactic acid biosensor. We noticed that the present biosensor has wide linear detection range of concentration from 1 × 10^−4^–1 × 10^0^ mM with acceptable sensitivity about 41.33 ± 1.58 mV/decade. In addition, the proposed biosensor showed fast response time less than 10 s, a good selectivity towards l-lactic acid in presence of common interfering substances such as ascorbic acid, urea, glucose, galactose, magnesium ions and calcium ions. The present biosensor based on immobilized ZnO nanorods with lactate oxidase sustained its stability for more than three weeks.

## 1. Introduction

Semiconductor metal oxide nanostructures based nanosensors have wide applications in biological, environmental and analytical chemistry sciences [1–4]. Especially one dimension nanostructures have promising applications in optics, optoelectronic, sensors, and actuators because these low dimensional structures exhibit attractive semiconducting, piezoelectric and pyroelectric properties, *etc.*, as described in the literature [1,4–6]. The properties of these metal oxide nanostructures such as fast electron communication, high surface to volume ratios and electro-catalytic effectiveness make them suitable matrices for effective immobilization and the desired transducing phenomena. Moreover, electrochemical enzyme-based transducers are attractive due to their ease in detection of target analytes (enzymes and proteins), high sensitivity, low cost, simplicity and low power consumption [7]. Recently, the research trends towards nanostructures of electrochemical sensors based on various materials have increased. ZnO has attracted more attention among researchers for sensing bioactive substances due to its two well-known features, namely a wide band gap (3.37 eV) and high exciton binding energy (60 meV). The structure of ZnO is tetragonal, in which Zn^2+^ and O^2−^ ions are alternatively arranged along the c-axis [8]. The ZnO material has two planes in its structure with opposite polar faces and surface relaxation energies. These nanostructures have relatively same size to those of bioactive chemical species to be sensed and are potential transducer candidates in generating electrical signals. Furthermore, ZnO has high ionic bonding characteristics (60%) and sustains its stability for a long time at biological pH values. The nanostructures of ZnO show strong bonding with enzyme/ionophore membranes due to their high ionic properties. All these properties of ZnO enhance the efficiency of ZnO nanosensors with respect to output signal, catalytic effects and facilitate the ease of flow of testing substance through the sensors. The high surface to volume ratios of ZnO nanorods support their use as strong candidates in the area of nano-chemical sensors [9,10]. ZnO nanorods are n-type semiconductors and their electrical transport dependency is ascribed to the adsorption/desorption properties of the chemical species [11–16]. A variety of one dimensional (1D) ZnO nanostructures such as nanorods, nanowires and nanotubes *etc*., have been synthesized by different techniques and these nanostructures have been employed in making of many nanodevices such as electric field-effect switching [17], single electron transistors [18], biological and chemical sensing [19] and luminescence [20] *etc.* Among these nanostructures, ZnO nanorods are widely used in the field of biosensing due to their high surface to volume ratios [21–24].

The isoelectric point (IEP) of lactate oxidase is 4.6 and that of ZnO is (9.5), this large difference in IEP between ZnO and lactate oxidase is evident for the ZnO nanomaterial to behave as a better material to immobilize low IEP proteins or DNA through strong electrostatic bonding [25–27]. Lactic acid biosensors are in high demand in clinical analysis, biotechnology, food industries and sports for the quick determination of lactic acid [28]. Amperometric biosensors commonly use enzymes including NAD^+^-dependent lactate dehydrogenase [29–32], hydrogen peroxidase, lactate oxidase [32–35] and flavocytochrome b_2_ [37–39] for lactate determination. The purpose of all these enzymes is to provide the simple pathway for the oxidation of l-lactic acid to pyruvic acid. Lactic acid is the final product of anaerobic glycolysis; due to this lactate level in blood is essential a metabolic sign for availability and magnitude of anaerobic glycolysis [40,41]. Because of the anaerobic glycolysis these phenomena arise in the pathophysiology of hypoxia, anoxia, or ischemia. When the H^+^ ion concentration inside the cell increases due to the above conditions, the intracellular pH decreases and the resulting acidosis decreases myocardial contractility as well as changes the impulse propagation. The enzyme system of many cells is also affected by pH changes, so for this reason the cell itself has developed plenty of ion channels, pumps, exchangers, and transporters to control the optimum intracellular pH. The exaltation of lactate anion and dissemination of lactic acid seems very valuable in the mechanics of monitoring the intracellular pH in these circumstances [42,43]. Yet the cellular mechanics for controlling the intracellular pH are not well realized in metabolic stress situations. This is because no any particular method yet is developed to measure the extracellular lactate [44]. The determination of the amount of lactic acid in the food industry is also crucial, especially in investigating the quality of dairy products and their improvement.

The presence of amounts of lactic acid in food products has a great impact on the stability, flavor, and storage life time. Many methods had been tested for detection of lactate such as methods for chemically oxidizing lactate, spectrophotometric detection of acetaldehyde and chromatographic methods. Moreover, other techniques use the enzyme kits or biosensors involving enzymes in the detection of lactate and in doing so these techniques have replaced the chemical methods in many clinical and analytical laboratories. Many amperometric lactate biosensors have been developed for more than 20 years, in order to analyze real samples, but these amperometric biosensors have some limitations with respect to intracellular lactate determination [45–47]. On other hand the potentiometric technique has the advantage of monitoring the intracellular pH as well as measuring the concentration of lactate.

In the present work, we have developed for the first time an independent potentiometric biosensor based on immobilized ZnO nanorods with lactate oxidase. The proposed biosensor showed linear responses to lactic acid concentration, high selectivity, better sensitivity and storage stability.

## 2. Experimental Section

### 2.1. Materials

l-Lactate-oxidase from *Pediococus* species (E.C. 1.1.3.2, activity 40 U·mg^−1^), l-lactic acid, glutaraldehyde (crosslinking molecule), zinc nitrate hexahydrate [Zn(NO_3_)_2_·6H_2_O], hexamethylenetetramine (HMT), d-glucose, l-glucose-fructose were purchased from Sigma Aldrich (Sweden). Phosphate buffer solution (PBS) was prepared by mixing the specific concentration of each of the following chemical compounds in deionized water: potassium chloride (KCl), sodium chloride (NaCl), potassium hydrogen phosphate (K_2_HPO_4_), sodium dihydrogenphosphate (NaH_2_PO_4_), calcium chloride (CaCl_2_) and the pH of solution was adjusted by using 100 mM hydrochloric acid (HCl) and 100 mM sodium hydroxide (NaOH); all these chemicals were also bought from Sigma Aldrich. Other than these all chemicals used were of analytical grade.

### 2.2. The Fabrication of ZnO Nanorods on Gold Coated Glass

**Figure 1.**
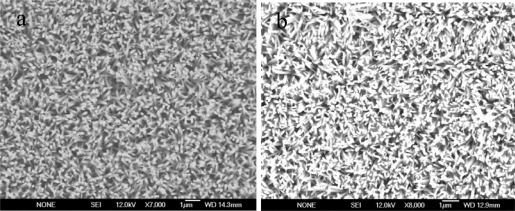
(**a**) The (FESEM) image of ZnO nanorods grown on gold coated glass substrate using hydrothermal growth method. (**b**) The (FESEM) image of lactate oxidase immobilized ZnO nanorods.

The fabrication of ZnO nanorods on gold coated glass substrates was as follows: firstly glass substrates were cleaned with isopropanol in an ultrasonic bath for 15 min, and then washed with deionized water and dried by nitrogen gas. After that glass substrates were fixed into the vacuum chamber of a Satis CR 725 evaporator instrument, for the deposition of thin film of 20 nm of titanium as an adhesive layer, followed by a layer of 100 nm thickness of gold. The growth of ZnO nanorods on these substrates was carried out by using the low temperature growth method (aqueous chemical growth method). The process of growing ZnO nanorods was as follows: first of all the gold coated substrates were washed with deionized water and dried by nitrogen gas, afterwards a homogeneous layer of zinc acetate was deposit on those gold coated substrates using the spin coating technique. The purpose of using the zinc acetate layer was to produce nucleation sites on the surface in order to grow well aligned ZnO nanorods of controlled length. The substrates with seed layer were annealed in an oven for 15 min at 130 °C and then affixed onto the Teflon sample holder and dipped into an equimolar solution of zinc nitrate and hexamethylenetetramine, then kept inside the oven below 100 °C for 5 to 7 h. When the growth time was finished, these substrates were then washed with deionized water and dried by nitrogen gas. The morphological study of grown ZnO nanostructures was carried out by using field emission scanning electron microscopy (FESEM) and we found that well aligned and controlled in length ZnO nanorods were grown on the gold coated substrates, as shown in Figure 1(a).

### 2.3. Immobilization of ZnO Nanorods with Lactate Oxidase

In this part of the experimental work, we developed five biosensor electrodes based on ZnO nanorods by immobilizing with lactate oxidase and crosslinking molecule (GA). We prepared 2.5% GA solution in 0.1 mM phosphate buffer solution (PBS) as well as lactate oxidase solution in PBS having a concentration of 2 mg/mL of enzyme. After mixing GA and enzyme solution in one bottle, we dipped the ZnO nanorods electrodes into it for different times in order to make ensure the saturation of the ZnO nanorod surface with the monolayer of enzyme. We found that after a time of 5 min, a complete enzyme monolayer was physically adsorbed on the surface of ZnO nanorods as shown in Figure 1(b) and therefore we followed this immobilization timing for all experiments. Ellipsometric measurements were performed to confirm the thickness of the layer of enzyme on the surface of the ZnO nanorods, which was about 100 ± 0.14 nm. When a sensor electrode was dipped into the solution of enzyme for 5 min, then the thickness of the enzyme monolayer was found to be 3 nm. The potentiometric response of the present biosensor electrode based on immobilized ZnO nanorods was as determined as follows: the immobilized ZnO nanorods electrode was taken as working electrode and silver-silver Ag/AgCl as a reference electrode and the (EMF) output response was measured using a Metrohm pH meter (model 827) and a Keithley (2400) is the time response measurement instrument and that was employed for the measurement of the time response. The biosensor electrodes were kept at 4 °C when not in use.

## 3. Results and Discussions

**Figure 2.**
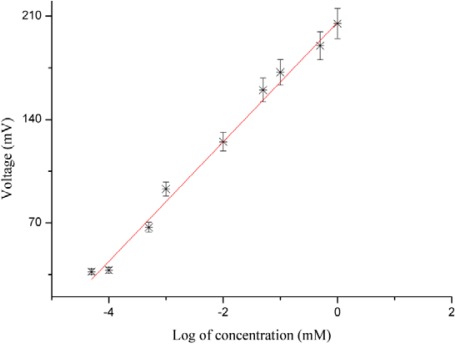
Calibration curve of lactate oxidase immobilized ZnO nanorods biosensor for l-lactic acid concentration 1 × 10^−4^ to 1 × 10 ^0^ mM.

The electrochemical response (EMF) of our proposed l-lactic acid biosensor based on lactate oxidase immobilized on the surface of ZnO nanorods in test electrolyte solution is shown in Figure 2.

During the investigations, the output response of the biosensor was changing with the change in l-lactic acid concentration in the test solutions. Because during the oxidation of l-lactic acid in the presence of immobilized lactate oxidase, unstable pyruvic acid was produced into the solution and this pyruvic acid was spontaneously converted into pyruvate and H_2_O_2_, as given in Equation (1):

(1) $$L-\text{lactic acid}\;+\;O_{2}\overset{{\text{LO}}_{x}}{\to }\text{Pyruvic acid}\;+\;H_{2}O_{2}$$

It is the concentration of charges which is responsible for the output response of the proposed l-lactic acid biosensor. This observed potential was mainly due to the presence of charges that varied around the working electrode [48]. The output response of l-lactic acid is also dependent on the potential catalytic activity of the lactate oxidase in the presence of oxygen, with more catalytic activity resulting higher output response. When we tested the proposed biosensor electrode in different l-lactic acid concentrations ranging from 1 × 10^−4^ to 1 × 10^0^ mM, the biosensor showed a linear output response for that concentration range, as shown in Figure 2. The sensitivity of the present biosensor was observed to be around 41.33 ± 1.58 mV/decade.

### 3.1. Effect of pH and Temperature on the Performance of Biosensor

The surroundings also have a significant effect on the output response of a biosensor when it is used. For the examination of the pH effect on the output response of the proposed biosensor, we tested the biosensor electrode in lactic acid solution in PBS at different pH values ranging from pH 3 to 12. We observed that the biosensor showed a gradual increase in the response for pH values 6 to 9. However, the biosensor had shown a decreasing trend for below pH 6 and above the pH 9. The decreasing response of biosensor in acidic medium might be due to the slow dissolution of ZnO nanorods in acidic as well as in alkaline media [49, 50]. This is the reason we carried out all experiments in PBS of pH 7, which is very close to the biological pH value of 7.3. Moreover it is kinetically favorable for enzyme based reactions to work at higher pH [51].

**Figure 4.**
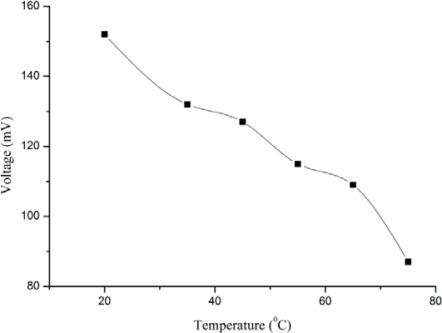
Calibration curves showing the study of EMF response with the change in temperature values.

The electrochemical response (EMF) of a biosensor also depends on the ambient temperature. For this purpose, we performed the controlled experiments at temperatures ranging from 23 °C to 75 °C. During the investigation, we observed that the present biosensor worked very well at room temperature rather than at high temperature, as shown in Figure 4. It has been reported in the literature that the lactate oxidase has its optimum activity at 30 °C [52], but the response of the present biosensor was found higher at room temperature due to possibly enhanced activity of the enzyme on the surface of ZnO nanorods and might be the ZnO behaved as a promoter for lactate oxidase.

### 3.2. Study of Common Interfering Substances on the Output Response of l-Lactic Acid and Response Time

Selectivity of the biosensor is one of the fundamental parameters for the performance evaluation and it was evaluated in the test electrolytic solution in the presence of other interfering species. During this experimental part, we studied the most commonly interfering species such as ascorbic acid, l-cysteine, glucose, urea, magnesium ions (Mg^2+^), and calcium ions (Ca^2+^), using a separation method for observing the biosensor selectivity.

**Figure 5.**
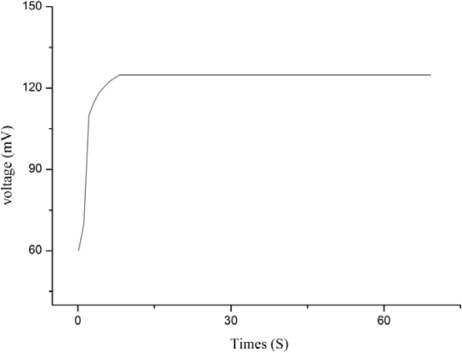
The response time of the proposed biosensor.

The calculated selectivity coefficient (K) values for these interfering substances are given in Table 1. It has been seen already that ascorbic acid has a significant effect on the response of lactic acid biosensors [53], but the present biosensor has shown no response to ascorbic acid. We used the 1 × 10^−3^ mM concentration range of each interfering substance. It has been observed that the proposed biosensor has the ability to work very well against the common interfering substances and therefore can be applied to determine the l-lactic acid in biological fluids, food, sport and clinical samples. Beside the selectivity, we examined the response time of the biosensor for all the detectable concentration ranges. It was observed that biosensor showed fast response times for higher concentrations and slower response times for low concentrations. Thus, the biosensor has shown a response time less than 10 s for all concentrations ranging from 0.01 mM to 1 mM, as shown in Figure 5.

### 3.3. Study of Life Time of Biosensor and Reproducibility

This study demonstrated that the biosensor has a lifetime stability of more than three weeks and also good reproducibility. We tested the lactic acid biosensor based on lactate oxidase immobilized on ZnO nanorods for three consecutive weeks and found that the biosensor maintained the detection range of lactic acid concentrations but in the third week a small decrease in sensitivity was observed as shown in Table 2. This might be due to the separation of lactate oxidase from the crosslinking molecules (GA).

### 3.4. Comparison of Present Proposed Biosensor with Already Existing l-Lactic Acid Sensors

Table 3 shows the results of the proposed l-lactic acid compared with the already existing l-lactic acid sensors. The proposed sensor based on the lactate oxidase immobilized on the ZnO nanorods has shown a low detection limit for the lactic acid, fast response time, good storage stability, better sensitivity, and high selectivity against interfering substances. These all advantageous features prove that the proposed biosensor based on the ZnO nanorods has the ability to firmly bind enzyme molecules on its surface and exhibited a selective determination of the target analyte.

## 4. Conclusions

In the present work, we have successfully demonstrated the potentiometric determination of l-lactic acid using ZnO nanorods immobilized with lactate oxidase in conjunction with (GA) crosslinking molecules by using a physical adsorption method. The proposed biosensor showed good stability, linearity, sensitivity, and selectivity when it was exposed to l-lactic acid test solutions. Based on all obtained results, the proposed sensor can be applicable to detect l-lactic acid in drugs, food and other biological samples.

## Figures and Tables

**Figure 3.**
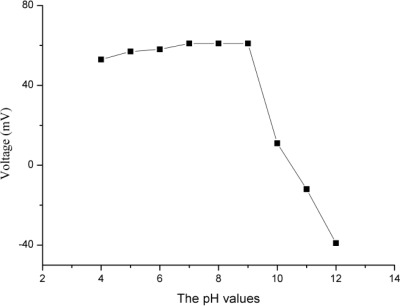
Calibration curves showing the study of EMF response with the variation of pH values.

**Table 1.** Selectivity coefficient in 10^−3^ mM.

**Interference (B)**	$\mathrm{Log}\;K_{\mathrm{lactic acid},\;B}^{\mathrm{pot}}$
**Mg^2+^**	0.5323
**Ca^2+^**	0.7259
**Glucose**	1.0404
**Urea**	1.3791
**l-cysteine**	1.1856
**Galactose**	1.5485
**Vitamin C**	1.7421

**Table 2.** The life time of electrode.

**Number of days**	**Slope (mV/decade)**	**Linear range (mM)**
**1**	41.33 ± 1.58	1 × 10^−4^–1 × 10^0^
**7**	42.57 ± 1.82	1 × 10^−4^–1 × 10^0^
**14**	41.39 ± 1.72	1 × 10^−4^–1 × 10^0^
**21**	40.80 ± 1.84	1 × 10^−4^–1 × 10^0^

**Table 3.** Comparison of lactose biosensor with the previous work.

**No.**	**Slope (mV/decade)**	**Times respond (s)**	**Detection Limit (mM)**	**Linear range (mM)**	**Lifetimes**	**Reference**
**1**	-	90	-	5.0 × 10^−2^–25 × 10^−2^	2 weeks	[29]
**2**	-	40	1.0 × 10^−1^	5.0 × 10^−1^–6.0 × 10^0^	2 weeks	[30]
**3**	24 ± 2	15	1.0 × 10^−1^	1.0 × 10^−1^–1.0 × 10^1^	-	[31]
**4**	-	30	1.0 × 10^0^	1.0 × 10^0^–15 × 10^0^	-	[32]
**5**	-	-	1.0 × 10^−3^	2.0 × 10^−4^–8.0 × 10^−3^	2 weeks	[34]
**6**	-	-	-	Up to 1.6 × 10^1^	15 days	[35]
**7**	-	30–42	1.0 × 10^−2^	1.0 × 10^−2^–1.0 × 10^0^	3–9 days	[37]
**8**	-	-	1.0 × 10^−3^	1.0 × 10^−3^–1.0 × 10^1^	5 weeks	[38]
**9**	41 ± 2	≈10	1.0 × 10^−4^	1.0 × 10^−4^–1.0 × 10^0^	More than 3 weeks	this work
